# DNA bridges: A novel platform for single-molecule sequencing and other DNA-protein interaction applications

**DOI:** 10.1371/journal.pone.0260428

**Published:** 2021-11-22

**Authors:** Maurizio Righini, Justin Costa, Wei Zhou

**Affiliations:** Department of Advanced Research and Development, Centrillion Technologies, Palo Alto, California, United States of America; The Chinese University of Hong Kong (Shenzhen), CHINA

## Abstract

DNA molecular combing is a technique that stretches thousands of long individual DNA molecules (up to 10 Mbp) into a parallel configuration on surface. It has previously been proposed to sequence these molecules by synthesis. However, this approach poses two critical challenges: 1-Combed DNA molecules are overstretched and therefore a nonoptimal substrate for polymerase extension. 2-The combing surface sterically impedes full enzymatic access to the DNA backbone. Here, we introduce a novel approach that attaches thousands of molecules to a removable surface, with a tunable stretching factor. Next, we dissolve portions of the surface, leaving the DNA molecules suspended as ‘bridges’. We demonstrate that the suspended molecules are enzymatically accessible, and we have used an enzyme to incorporate labeled nucleotides, as predicted by the specific molecular sequence. Our results suggest that this novel platform is a promising candidate to achieve high-throughput sequencing of Mbp-long molecules, which could have additional genomic applications, such as the study of other protein-DNA interactions.

## Introduction

Next generation sequencing (NGS) methods are often used for whole-genome sequencing, and these methods require the large genome to be fragmented into relatively short DNA molecules (≈500 bp) for sequencing. These fragments are then amplified and sequenced, producing a collection of short reads. These short reads are then computationally analyzed by either mapping to a reference genome [1] or *de novo* assembly [2] to reconstruct the complete sequence of the original genome. However, NGS technology has certain limitations, i.e., difficulty in analyzing repetitive regions in the genome, often requiring a reference sequence for the alignment of the short reads, and limited ability for full *de novo* genome assembly [3]. Additionally, NGS methods typically require an amplification step, which introduces sequence-dependent biases and loss of epigenetic information in the form of methylcytosine [4].

These technical limitations have been partially overcome by third-generation sequencing (TGS) technologies [5]. Although these methods have their own limitations, the key advantage of TGS methods over NGS is that they can sequence much longer molecules and do not require an amplification step. Current TGS technologies can obtain read lengths in the tens to hundreds of kilobases [6].

Molecular combing is a technique that uses the motion of an air-liquid interface to extend thousands of long DNA molecules in a parallel configuration onto a surface [7–11]. The combing of DNA molecules up to 12 Mb long has been reported [12]. DNA combing is typically used for optical mapping of long DNA molecules [13]. Optical maps can reveal the spatial locations of sequences of interest along the length of the molecules, for example, by the hybridization of fluorescent probes [14–21]. However, this approach cannot provide information beyond the location and distance between the target sequences. Most assays that employ molecular combing rely on hybridization as a read out, while more complex biochemical assays that involve enzymes are relatively sparse in the literature.

DNA replication [22–24] and RNA transcription [25–27] have been performed on combed DNA molecules. However, these assays suffer two major roadblocks. First, the combed molecules are known to be overstretched (i.e., longer than the natural B-form contour length), which has been shown to hinder enzymatic activity [25, 28]. Second, the combing substrate itself, sterically blocks enzymatic access to the DNA backbone [23].

For instance, optical sequencing of combed molecules through DNA polymerase synthesis was attempted by the Schwartz laboratory almost two decades ago [23] but has never become a reality. Unsurprisingly, in all sequencing methods, the enzymatic reactions operate on DNA molecules that are either free in liquid or tethered at the 5’ end. For instance, in NGS methods, DNA molecules are attached to a solid surface (a bead or a slide), while in TGS, they are forced to pass through a pore or a nanowell. However, under no circumstance are the sequenced molecules under significant tension or resting immobilized on a surface.

Moreover, when fluorescent probes are dispensed to combed DNA molecules over multiple cycles, as in optical sequencing, the background noise on the surface increases over time and eventually disrupts the sequencing process [23].

Previous reports have demonstrated that decreasing surface tension at the combing air-liquid interface can relax the strain along combed DNA [25]. Alternatively, anchoring DNA to the surface through DNA binding proteins [27] or by using an intermittent pattern of hydrophilic and hydrophobic regions on the stretching substrate can also avoid DNA overstretching [24, 29]. Despite these efforts, it is not currently possible to precisely tune the stretching, i.e., the tension of the combed DNA molecules.

The second challenge, i.e., the surface problem, has never been addressed in molecular combing studies. This is not surprising since the principle of molecular combing requires a surface to comb the molecules.

Here, we demonstrate a novel platform for combing thousands of long DNA molecules with a tunable stretching coefficient. The combing substrate can further be spatially dissolved in defined patterns, leaving the combed molecules suspended like bridges across a series of micrometer-sized canyons. The genomic DNA in these regions exists in a fully aqueous environment across the spans, permitting enzymatic access to the template. This technique eliminates background fluorescence, makes the nucleic acid backbone freely accessible and enables tuning of the molecular tension. These features make this configuration promising for protein-DNA studies and for the robust optical mapping and sequencing of megabase-long molecules.

## Results

### Preparation of DNA bridges

**Fig 1:**
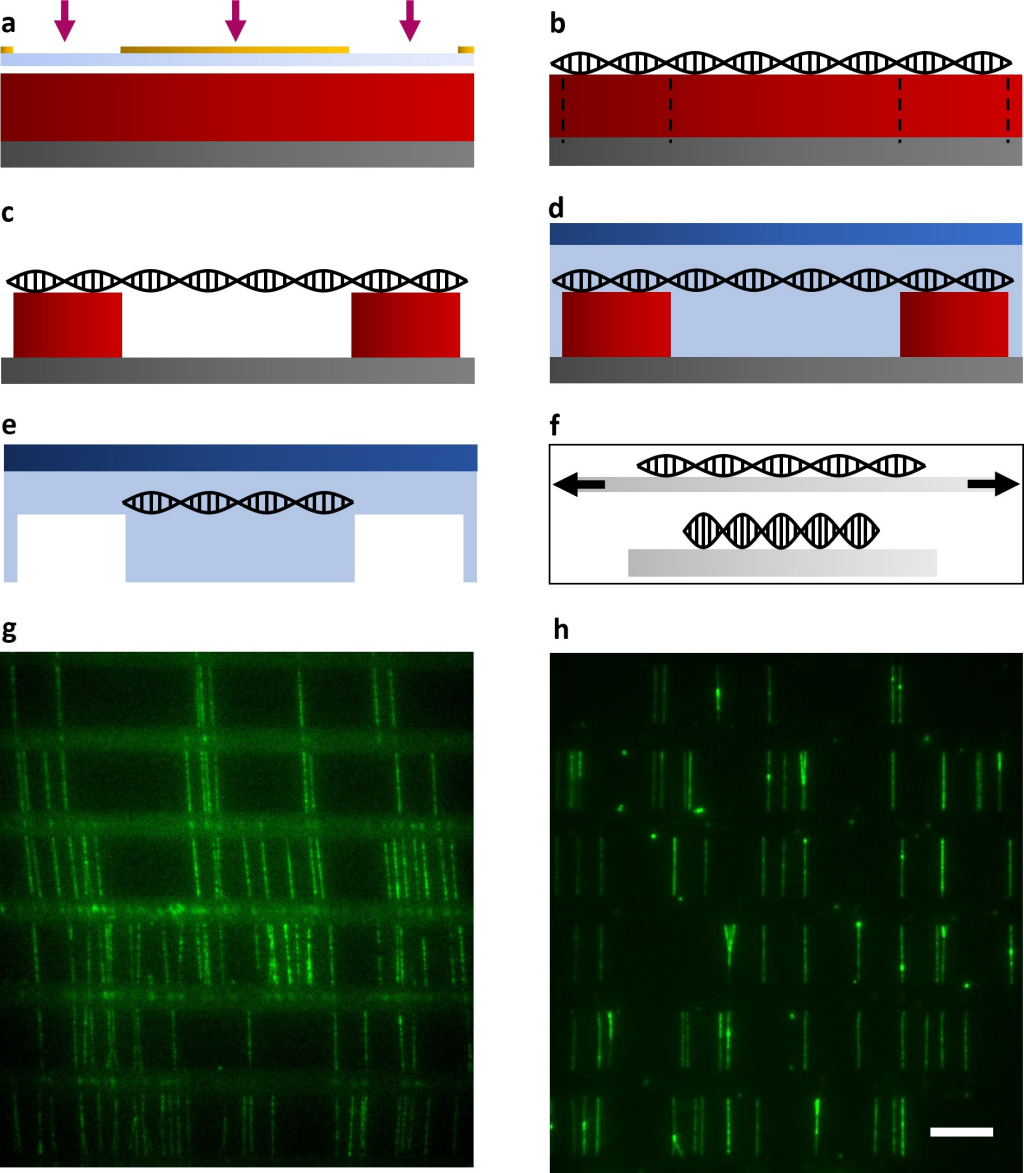
DNA bridge preparation and fluorescence images of the sample. a) Schematic of a silicon substrate (gray) coated with SU-8 (red), illuminated by UV light through a transparent mask (light blue) patterned with chromium (yellow). b) DNA was combed on the SU-8 layer after selective crosslinking. c) The unexposed portions of SU-8 were dissolved, leaving the DNA molecules suspended between parallel SU-8 features. d) We cast a gel (light blue) onto the sample and covered it with a treated quartz slide (dark blue). e) After separation of the substrates, the gel remained attached to the quartz slide, capturing the suspended portions of the molecules. f) DNA was combed on a stretched piece of PDMS (top). When the PDMS was relaxed, the combed molecules decreased in extension (bottom). g) Fluorescence image of the sample described in Fig 1C, seen from above. h) DNA molecules that were transferred into a hydrogel as described in Fig 1E. The scale bar (white) is 10 μm long and refers to both Fig 1G and 1H.

A spin-coater was used to deposit an ≈2 μm layer of epoxy-based negative photoresist SU-8 onto a silicon substrate. After a soft bake on a hot plate, the SU-8 substrate was selectively exposed to UV light through a photomask (Fig 1A). The mask pattern exposed a series of equally spaced parallel lines with the same width.

The proper combination of these two parameters (width and distance) can be chosen according to the desired application. After postexposure baking, the substrate (undeveloped) was ready for DNA capture. SU-8 has been previously shown to be an effective substrate for DNA combing [30].

The substrate was immersed into a cuvette that contained genomic DNA molecules diluted in a combing buffer. The DNA concentration and incubation time determine how much DNA is captured on the SU-8 surface. The sample was oriented so that the lines exposed through the mask were parallel to the liquid-air interface. A controlled vertical retraction of the substrate from the cuvette fixed the DNA molecules in a linear arrangement perpendicular to the UV-exposed lines on the SU-8 surface (Fig 1B).

Next, the sample was treated with propylene glycol methyl ether acetate (PGMEA) to selectively remove any SU-8 that was not exposed to UV light through the mask. Every molecule that had at least one attachment point on adjacent lines of the pattern remained suspended, like a bridge, between two SU-8 features (Fig 1C). The sample was washed with isopropanol and rinsed with water.

DNA molecules were then stained with the intercalating dye YOYO-1 and illuminated by 488 nm laser light. The sample was imaged under a custom-built inverted TIRF (total internal reflection fluorescence) microscope (Fig 1G). SU-8 is visible (horizontal lines) due to its autofluorescence. The background fluorescence around the suspended DNA was very low due to the distance of the DNA from the bottom surface (≈2 μm), which was not reached by the total internal reflected illumination. The DNA was suspended in aqueous solution and was accessible from any direction.

A few DNA molecules appeared to bifurcate in a “Y” shape, indicating that two or more molecules were entangled together.

### Transfer of the molecules to a hydrogel

Combed DNA molecules break when the liquid on the substrate evaporates. Additionally, prolonged illumination of DNA molecules stained with dimeric dyes leads to photocleavage [25, 31], and under high-intensity illumination, we observed DNA molecules breaking. Once a DNA molecule breaks, it retracts to a random coil (S1 Fig).

To prevent the DNA from breaking, we casted a thin layer of acrylamide gel over the sample, and a bind-silane-treated quartz slide was placed on top of the gel to spread the gel evenly over the SU-8 surface (Fig 1D). After polymerization of the gel, we separated the substrate and the slide. The hydrogel, which remained attached to the treated surface, tore away the regions of DNA that were suspended between the SU-8 structures, and these regions of DNA were captured into the gel. The DNA regions that were attached to the SU-8 surface remained attached to it and did not transfer (Fig 1E). Therefore, all the DNA fragments transferred to the gel had the same length, which we could tune by varying the distance between the features of the photomask. In addition to preventing the DNA molecules from recoiling when broken, this configuration eliminated the autofluorescence of SU-8 (Fig 1H). It is also important to note that the molecule spacing or density can be controlled by reducing the DNA concentration during the combing step. Reducing the density makes it easier to distinguish which consecutive fragments belong to the same molecule.

Thousands of highly ordered 5 μm long DNA fragments were previously shown by Guan and coworkers on a micropatterned PDMS surface [32]. However, in our approach, the DNA fragments were longer (up to 40 μm) and embedded in the gel rather than attached to the surface.

Using our approach, the DNA molecules remained stable and did not move over time (>24 h.) when kept in an aqueous solution on a shaker.

### Tuning the molecular extension

The process of DNA combing stretches DNA molecules to a length that is greater than their natural contour length (0.34 nm x number of bp). Enzymatic activity has been shown to be negatively impacted when DNA molecules are overstretched [25, 28]. To circumvent this problem, we developed a method to control the stretching of combed DNA molecules. First, we cast a thin layer of polydimethylsiloxane (PDMS) on a clean surface. Once cured, PDMS is a relatively elastic material. Next, we immobilized two ends of the PDMS polymer onto the clamps of a custom-built mini vise. We then extended the vise to stretch the PDMS to the desired length. Finally, we deposited a drop of the combing solution onto the stretched PDMS and used the rolling drop method [33] to comb the DNA molecules onto the hydrophobic surface. We dragged the drop in the same direction in which the PDMS was stretched. This resulted in thousands of molecules aligned to the stretching direction. When we released the PDMS from the vise, the DNA molecules attached to it relaxed to a shorter length (Fig 1F). By controlling how much we extended the PDMS in the vise, we could precisely tune the final length of the molecules following relaxation.

The DNA molecules that are combed on the PDMS can be transferred to an SU-8-coated surface. SU-8 was coated on a silicon surface and exposed with the line pattern but not developed. The DNA molecules were transferred from the PDMS onto the SU-8 surface by contact printing [32, 34]. After development of the sample, we obtained relaxed DNA molecules suspended between SU-8 features. Observation of the suspended molecules indicated that most molecules transferred intact from one surface to the other.

The stretch of combed molecules has been shown to depend only on the characteristics of the surface and the surface tension at the air/liquid interphase [8, 35]. To measure the stretching factor, we used long-range polymerase chain reaction (PCR) to amplify a 10 kb region of lambda DNA. The PCR product was combed onto a PDMS surface, and the lengths of the molecules were measured. The length distribution showed a peak at approximately 3.76 μm, which is 111% of the predicted contour length for a 10 kb B-conformation DNA molecule (10 kb x 0.34 nm = 3.4 μm). Based on previously reported data [36], we estimate that the tension sustained by these molecules is approximately 60 pN. When we captured molecules on PDMS extended with the mini vise to twice its length and then let it settle and transferred the molecules onto SU-8, we measured a peak at 1.94 μm, corresponding to 57% of the contour length, or a force estimated < 5 pN.

### Incorporation of fluorescent nucleotides by synthesis

**Fig 2:**
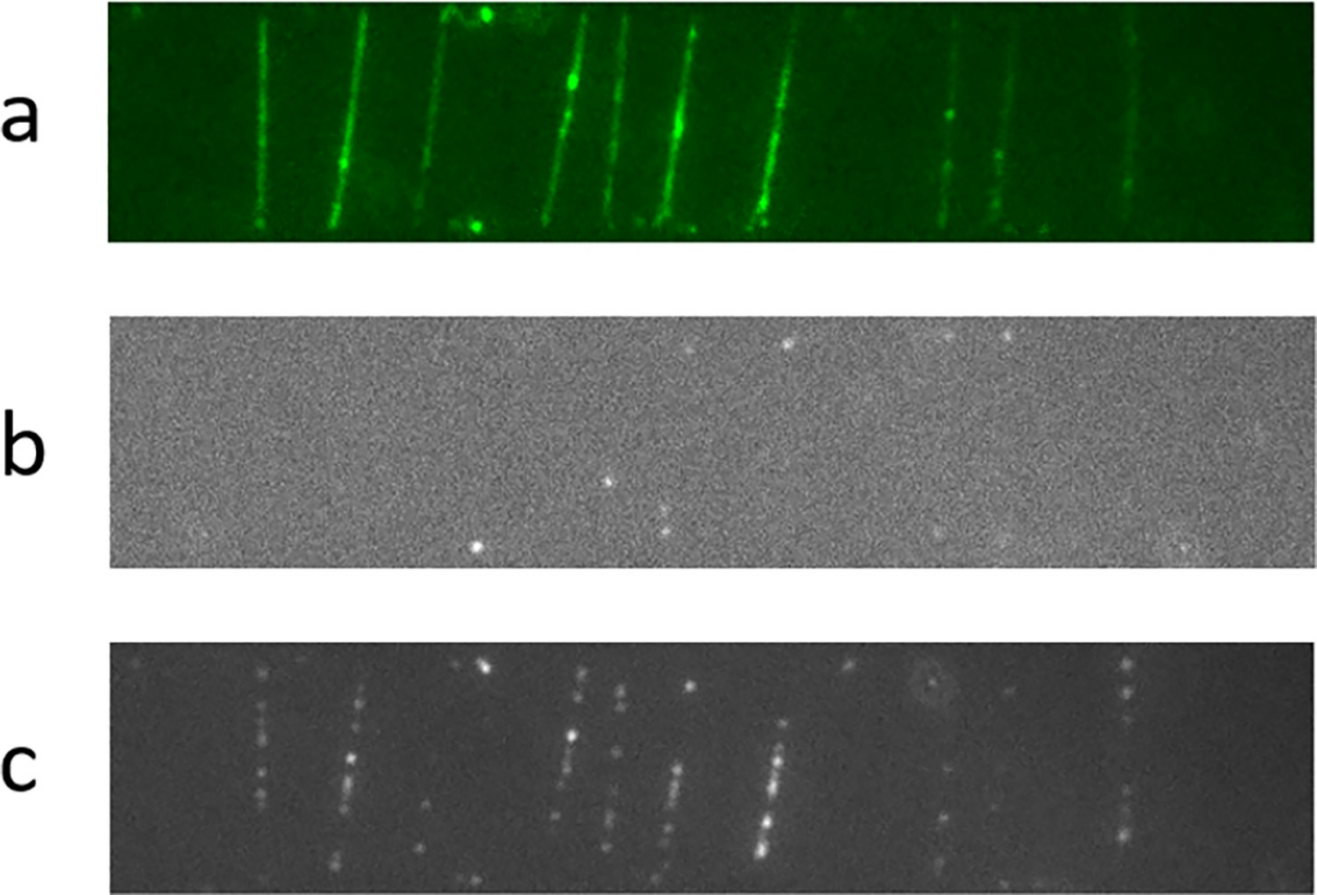
DNA bridges are enzymatically active. a) DNA molecules embedded in a gel and stained with the intercalating dye YOYO-1 were visualized and are rendered in green. b) A first incorporation reaction including polymerase, Alexa 647-labeled dUTP and natural dCTP, dATP and dGTP was carried out. An image of the sample was captured in the Alexa 647 channel. Only a few dots appeared, most likely due to elongation from random nicks. c) After incubation with a nicking enzyme, the previous incorporation reaction was repeated. This time, many labeled nucleotides were incorporated and could be observed along the molecules.

We demonstrated that the trapped molecules are enzymatically accessible by using DNA polymerase to incorporate labeled nucleotides. We used the Klenow fragment (exo-) in combination with Alexa Fluor 647-aha-dUTP and natural dATP, dCTP, and dGTP. In the first experiment, we poured a solution containing the enzyme and the four nucleotides onto the surface of the gel in which the DNA molecules were trapped and stained with YOYO-1 (Fig 2A).

After the reaction, we captured an image of the sample in the appropriate channel to visualize Alexa 647. We detected only a few fluorescent ‘dots’, and their locations lined up with the molecules (Fig 2B). Since there are no free 3’ OH groups for the initiation of synthesis along an intact DNA molecule, we believe that these incorporations occurred at random nicks. The Klenow fragment (exo-) can extend the 3’ hydroxyl terminus by displacing the strand downstream of the nick. Nicks might be present in the original DNA or might be caused by a step in our sample preparation, such as the transfer of the DNA molecules from PDMS to SU-8. Subsequently, we incubated the sample with a nicking enzyme, Nb.BtsI, that nicks the molecules at the sequence 5’…NNⱽCACTGC…3’ between the second N and the first C. We then repeated the same incorporation reaction that we already performed before nicking. This time, many luminous dots appeared on the molecules, indicating that the polymerase extended the new nicks, incorporating labeled nucleotides (Fig 2C).

**Fig 3:**
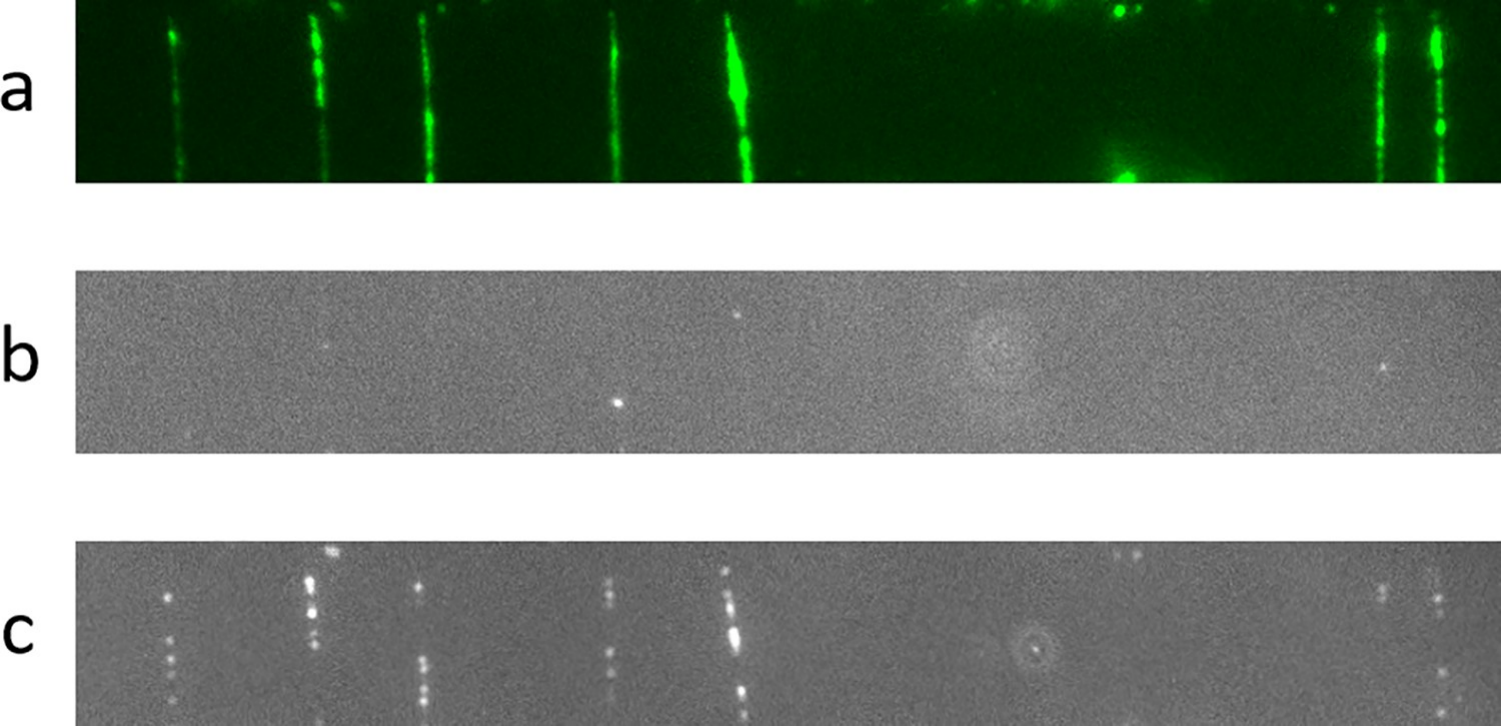
Selective incorporation of labeled nucleotides. a) DNA molecules embedded in gel were incubated with a nicking enzyme. The sequence downstream of the nick is CACTG. b) A first incorporation reaction, including only labeled dUTP and the polymerase, gave rise to only a few dots, presumably due to incorporation at random nicks preceding a T. c) An additional round of incorporation, including labeled dUTP and natural dCTP and dATP, was carried out. The labeled nucleotides were incorporated and appeared as fluorescent dots distributed along the length of the molecules.

In the second experiment, we demonstrated that labeled nucleotides are incorporated as predicted by the known DNA sequence. First, we incubated the sample with the nicking enzyme Nb.BtsI (Fig 3A). Since the recognition sequence is NNⱽCACTG, we know the bases that should be incorporated downstream of the nick, CACTGC. We first added the polymerase and labeled dUTP nucleotides. Since only a dCTP could be incorporated into the strand from the nick, dUTP was not incorporated. In Fig 3B, only a few dots are visible, presumably due to random nicks adjacent to a T base after the nick.

Finally, we repeated the same incorporation reaction as above, adding dCTP and dATP to the mix. This time, since these two nucleotides were present, labeled dUTP was also incorporated, and many fluorescent dots overlapped the DNA molecules (Fig 3C).

**Fig 4:**
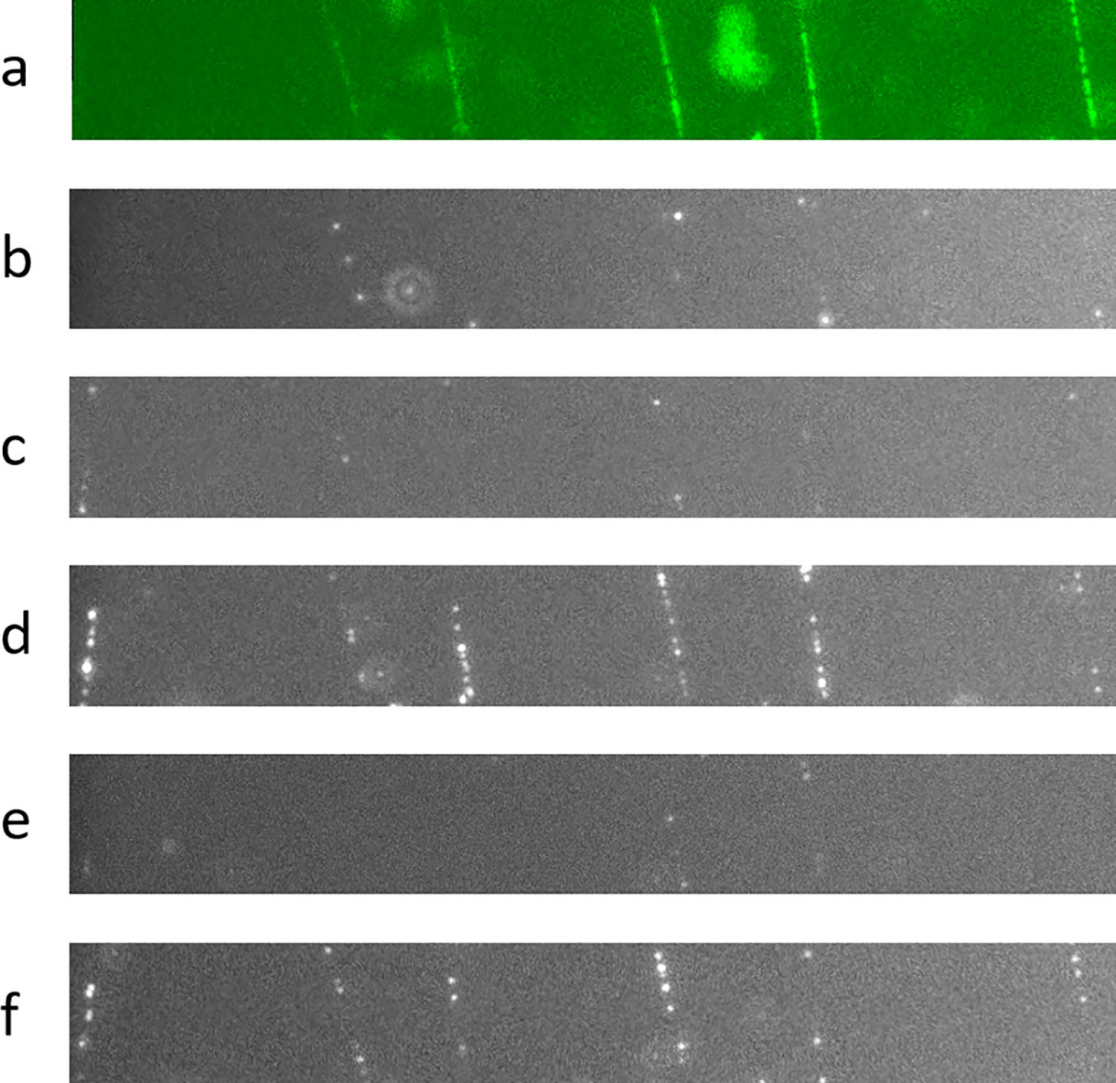
Selective incorporation of labeled nucleotides. a) DNA molecules embedded in gel were incubated with a nicking enzyme. The sequence downstream of the nick is CACTG. b) A first incorporation reaction was carried out with polymerase, dCTP and labeled dUTP. Only a few dots are visible. c) Second incorporation reaction with dATP and labeled dUTP. d) The same reaction shown in Fig 4B was repeated (dCTP and dUTP). This time, many dots appeared. e) The previous reaction (dCTP and dUTP) was repeated. f) Final incorporation reaction including dGTP, dATP, dCTP and labeled dUTP.

To prove even further that nucleotide incorporation is dictated by the sequence, we performed an experiment where we varied the combination of nucleotides added at each round of incorporation. We started by nicking the DNA (Fig 4A) with the same enzyme as in the previous experiment. During the first round of incorporation, we provided only dCTP and labeled dUTP. Since the sequence of incorporation is CACTG, dCTP was incorporated but not dUTP. The few dots visible in Fig 4B are once again attributed to random nicks.

For the second round of incorporation, we supplied dATP and labeled dUTP. Once again, dATP was incorporated but not dUTP (Fig 4C), and only a few dots appeared. In the third round of extension, we provided dCTP and labeled dUTP, as in the first reaction. However, in contrast to the first reaction, both dCTP and dUTP were now incorporated, and many fluorescent dots were visible (Fig 4A). Subsequently, we photobleached all the fluorophores in the field of view and repeated the previous incorporation, delivering dCTP and labeled dUTP again, and we observed only a few dots (Fig 4E). We speculated that these dots appeared at locations where the previous reaction had not been completed. In fact, 57% of these dots do not overlap with those in Fig 4D. The remaining 43% might also occur at new locations that were nevertheless too close to the previous locations to be distinguished. According to this interpretation, we can estimate that the first reaction was 83% complete or less.

Assuming a complete reaction after the second cycle, no more labeled dUTP nucleotides can be incorporated unless a dGTP is first added. Therefore, in the final incorporation cycle, we included dGTP, dCTP, dATP and labeled dUTP. Although we ignore the bases downstream of the recognition sequence, it is safe to assume that following G, more nucleotides are added until a new labeled dUTP is incorporated. In Fig 4F, we observe many dots along the molecules. Of these dots, only 73% superimpose with those of Fig 4D. The remaining 27% might be due to pre-existing nicks that were followed by at least one base G before any T. Their number is comparable to the number of dots observed in Fig 4B and 4C, which are presumably also due to pre-existing random nicks. Moreover, only 43% of dots in Fig 4D appeared again in Fig 4F. This is most likely due to the low efficiency with which a polymerase can synthetize from the 3’ end of a nucleotide labeled with a bulky fluorophore. This phenomenon is well documented in the literature [37–40].

## Discussion

Enzymatic activity on combed DNA molecules is hampered by two main limitations: combed DNA molecules are overstretched, and the surface itself sterically blocks free access to the nucleic acid backbone. Additional problems associated with traditional DNA combing platforms arise when multiple rounds of fluorescent probing are performed, such as for sequencing by synthesis applications, when the signal-to-noise ratio decreased due to the accumulation of nonspecific fluorescence background on the surface [23].

The method we have developed circumvents these problems by suspending thousands of individual combed DNA molecules in liquid or in a hydrogel with a tunable degree of stretching. The method is simple, inexpensive, and only requires a basic UV lithography setup.

To develop the SU-8 features, we submerged the substrate and the combed molecules in PGMEA for 1 minute. To our knowledge, there have been no reports that studied the effects of PGMEA on DNA, so there were concerns about the potential impact of this exposure on downstream enzymatic reactions. However, we did not observe any evidence to indicate that the DNA was compromised. Note that acetone (which can be used for long-term DNA preservation) also dissolves SU-8 and can be used as a substitute for PGMEA (S2 Fig).

To observe the DNA molecules suspended in the hydrogel, we stained the DNA with an intercalating dye (YOYO-1). Dyes in this family have previously been shown to interfere with enzymatic activity on DNA substrates [41], but we repeated our nucleotide incorporation assay without YOYO-1 staining with no observable difference. One explanation for this is that the DNA in our assay is labeled with a relatively low concentration of dye, so we cannot rule out that the enzymatic activity we observe is occurring in regions that have not been intercalated by a dye molecule. Furthermore, we have the option to carry out the DNA staining at the end of the experiment after the biochemistry of the assay is complete. However, for applications where fiducial alignment and registration of the DNA is required, this could be a limitation. Other DNA stains that target the minor groove and have been shown to have minimal enzymatic interference, such as DAPI (4’,6-diamidino-2-phenylindole), could also be used [42].

We have demonstrated that combed DNA molecules suspended in a hydrogel can be processed by enzymes. In the first experiment, we used both Klenow DNA polymerase (exo-) and the nicking enzyme Nb.Btsl. When we tried to incorporate labeled nucleotides without nicking the DNA first, only a few incorporations were observed because there were no free 3’ OH groups for the initiation of synthesis. We assume that the few spurious incorporations that we observed are most likely explained by the presence of random nicks caused by the sample fabrication procedure. When the DNA was treated with the nicking endonuclease, we observed a dramatic increase in fluorescent nucleotide incorporation along the DNA molecules. Note that the same experiment carried out on overstretched molecules led to a minimal number of incorporations. When performed on molecules combed on a surface, the same procedure did not show any incorporation at all.

After determining that the molecules are enzymatically accessible, we investigated whether it would be possible to apply sequencing-by-synthesis methods to the suspended DNA molecules. Although we could not determine the full sequence of the molecules in the absence of proper reagents, we demonstrated that modified nucleotides can be cyclically incorporated into the template according to the template sequence. For example, by using a nicking enzyme that cuts at a known restriction site, we know *a priori* that the sequence 3’ of the nick is CACTG. We leveraged this information to verify that when we used polymerase with varying combinations of labeled and unlabeled nucleotides, we observed proper incorporation of the correct nucleotide, as expected from the known template sequence at the nick.

After a first round of incorporation of labeled dUTP, we repeated the reaction a second time and observed a small number of incorporations, suggesting that the first labeled nucleotide addition in this system was ≤ 83% efficient. This observation suggests that multiple rounds of fresh reagents could be used to increase the incorporation rate.

Once we recorded the position of the fluorescent labels and photobleached them, we tried to resume extension and incorporate more labeled nucleotides. We found that only a fraction of the incorporation locations lit up again in a second cycle. The estimation of the fraction (43%) of labeled nucleotides that were extended is only approximate since we cannot distinguish multiple dots that overlap each other. This drop in the number of incorporations is not surprising because of the reduced efficacy of polymerases in extending from the 3’ end of a labeled nucleotide. We reason that the use of cleavable fluorophores should address this limitation.

Therefore, we envision sequencing DNA molecules in the DNA bridge configuration with the use of labeled nucleotides with cleavable fluorophores and reversible terminators [43]. It is noted that the random nicks in the DNA described above are as useful as enzymatically induced nicks, for sequencing. If successful, this method would enable read lengths 2 to 3 orders of magnitude longer than what is possible with current TGS techniques.

We anticipate two limitations to hinder the successful performance of sequencing-by-synthesis on DNA molecules suspended as described here.

When we transferred the DNA molecules into the hydrogel, the portions of the molecules that were affixed to the SU-8 surface remained attached to it and did not migrate (Fig 1E). For instance, Fig 1H shows consecutive pieces of DNA molecules missing approximately 33% of the original sequence. This proportion is dictated by the ratio of the distance between consecutive SU-8 features (the bridge length) to the thickness of the features itself. We can change this ratio to minimize the amount of nucleic acid that is lost. For instance, we have combed molecules over 5 μm features separated by 40 μm canyons, resulting in only an 11% loss of information. The ratio can be pushed further.

Second, we do not know which strand is nicked; therefore, we ignore the direction of the sequence detected. However, if sequencing is reiterated over hundreds of bp, it would be in principle possible to fit the point spread function to the fluorophore images in the first and last round of acquisitions and tell in which direction the polymerase has moved.

We envision the sequencing of large portions of Mb-long single molecules in a high-throughput configuration, where the distance between each sequence and their order on the molecule is known. On the other hand, even sequencing only a few bases following nicks would be sufficient to generate optical maps that are superior to those obtained from traditional optical mapping techniques. It is noted that once sequencing is completed it is possible to renick the molecules and sequence them again to validate the previous reads.

Our observations suggest that any damage caused to the DNA during the fabrication and gel trapping process is minimal, but further studies are needed to completely rule out any compromise of the suspended molecules.

Additional applications for this platform can be envisioned in protein-nucleic acid interaction studies [44–47] (S3 Fig). For instance, studies that examine the cooperative binding of multisubunit transcription factors would allow each of the proteins under investigation to be added individually to the system while being monitored by optical readout. Tagged enzymes such as DNA and RNA polymerases could be used to record template engagement in real-time, and kinetic data could be extracted from observations of their movement. As the system suspends thousands of genomic DNA in identical kilobase-length fragments, hundreds of interactions could be followed simultaneously in one field of view. As our platform combines the single molecule and optical benefits offered by molecular combing with a hydrogel system that facilitates enzymatic assays, we anticipate a variety of optical biochemical assays to complement many of the genomic technologies currently in use.

## Methods

### Substrate preparation

We diced a silicon wafer into squared pieces of 22x22 mm. We used one piece as a substrate and spin-coated it with 290 μL of SU-8 2002 (Microchem) at 500 rpm for 10 seconds and then at 1000 rpm for 30 seconds. We obtained a ≈2 μm thick layer of SU-8 on the silicon surface. We soft baked the sample at 95°C for 2 minutes on a hot plate. We used a mask aligner (ABM) to expose the surface to 365 nm UV light through a chromium photomask (Compugraphics), delivering approximately 80 mJ/cm^2^. The mask design is made of parallel lines with thickness varying between 5 and 10 μm. The space between the lines, coated with chrome, can vary between 5 and 40 μm. We then performed a postexposure bake at 95°C for 2 minutes.

### DNA combing

We introduced the SU-8-coated substrate vertically into a cuvette containing 1 mL of 0.5X MES buffer (pH 5.5) and approximately 40 ng of dispersed genomic DNA (Promega). We incubated for 1 hour and then retracted the substrate from the cuvette at 65 μm/sec. In the alternative procedure, we cast PDMS (Sylgard 184) mixed in a ratio of 10:1 elastomer to curing agent onto a clean surface. We baked it in the oven for 4 hours at 55°C. We cut a rectangular piece of PDMS (≈1 by 3 cm) and stretched it in a mini vise to twice its length. We dispensed 200 μL of 0.5X MES buffer (pH 5.5) containing approximately 40 ng of DNA on the surface. After one hour of incubation, we tilted the vise and dragged the drop with a razorblade (without touching the PDMS surface) in the stretching direction. We carefully relaxed the PDMS closing the vise. We then removed the PDMS and overlaid it on an SU-8 substrate prepared as described in the previous paragraph. We waited 20 minutes and then proceeded to separate the PDMS slowly from the SU-8 surface, detaching it in the direction of the DNA orientation.

### Sample development

We submerged the sample in a beaker containing approximately 50 mL of PGMEA and agitated it for 1 minute. We rinsed the sample in a second beaker containing approximately 50 mL of isopropanol for 10 seconds. Finally, we submerged the sample into a water bath and waited a few minutes until isopropanol was rinsed away. We removed the sample from the bath, keeping it horizontal, and ensured that its surface remained covered in water.

### Migration of the molecules into a hydrogel

We submerged quartz slides (22x22 mm) in a solution containing 94.4% ethanol, 5% acetic acid and 0.6% Bind-Silane. We incubated the substrates for 1 hour on a shaker. Finally, we rinsed it with water and blow-dried it with clean compressed air. We prepared 6% acrylamide/bis, 5% tetramethylethylenediamine (TEMED) and 219 mM ammonium persulfate (APS). We then mixed them in a solution with the following ratio: 90% acrylamide/bis, 5% TEMED and 5% APS. We dispensed 15 μL of the prepared solution onto the sample surface and quickly covered it with the bind-silane-treated slide. After approximately 20 minutes, the gel had polymerized, and we carefully detached the slide from the substrate with the help of a razor blade. We submerged the slide in water to hydrate the gel. To inspect the DNA molecules, we dispensed 50 μL of a 100 nM dilution of the intercalating dye YOYO-1 (Invitrogen) and incubated for 20 minutes at room temperature. Finally, we rinsed it with water.

### Incorporation of labeled nucleotides

We equilibrated the sample with 50 μL of 1x CutSmart buffer (NEB). We removed most of the liquid from the gel by touching it on the edge with absorbent paper. We then applied 50 μL of 1x solution of CutSmart buffer containing 10 units of the restriction endonuclease Nb.BtsI (NEB). We placed the sample in a humidified chamber and then in an oven at 37°C for 1 hour. We washed the sample in a water bath for 15 minutes. We equilibrated the sample with 50 μL of 1x NEB buffer 2. We removed the excess liquid. We then delivered 50 μL of 1x solution of NEB buffer 2 containing 10 units of Klenow (exo-) (NEB) and a combination of the four nucleotides depending on the experimental requirements (Alexa Fluor 647-aha-dUTP (Invitrogen), dCTP, dATP and dGTP (NEB)). Each nucleotide had a final concentration of 100 nM. We incubated the sample at 37°C for 30 minutes, washed the sample in 4x saline sodium citrate buffer for 20 minutes, and rinsed it with water.

### Imaging system

We built a through-objective inverted TIRF microscope. Two lasers (Coherent) emitting at 488 and 642 nm were combined into a single beam via dichroic beam splitters (Semrock). Total internal reflection was obtained through a 100X TIRF objective (Nikon). Fluorescence was collected by the same objective and passed through appropriate emission filters (Semrock) selected via a filter wheel (Thorlabs). Finally, images were collected with an EMCCD camera (Andor iXon).

## Supporting information

S1 Fig High-intensity illumination breaks DNA bridge molecules. Left (from top to bottom): sequence of breakage of an ensemble of molecules. Right (from top to bottom): sequence of breakage and recoiling of a single suspended molecule.
(TIF)

S2 Fig DNA bridges prepared with acetone. This sample was fabricated using acetone, instead of PGMEA, to dissolve the portions of SU-8 that were not crosslinked.
(TIF)

S3 Fig FRET can detect DNA-protein interactions. Förster resonance energy transfer (FRET) was previously used to monitor binding of a Cy5-labeled protein to nucleic acid stained with SYBR Gold [48]. We used DyLight 650 instead of Cy5, which is spectrally similar. First, we nicked the DNA bridges molecules with Nb.BtsI, then incubated the sample with biotin-11-dUTP, dATP, dCTP and polymerase. Finally, we incubated the sample with DyLight 650-labeled streptavidin. We captured an image of the sample (top) in the SYBR Gold channel. Then, we used 488 nm light to excite SYBR Gold and captured the image of the same molecules (bottom) with the emission filter (676/29) selected for DyLight 650. Note that SYBR Gold fluorescence leaks into the detection channel.
(TIF)
