# Supplementary figures and images for: DNA bridges: A novel platform for single-molecule sequencing and other DNA-protein interaction applications
Source: PLoS One. 2021 Nov 22;16(11):e0260428. doi: 10.1371/journal.pone.0260428 (PMC8608331; doi:10.1371/journal.pone.0260428)

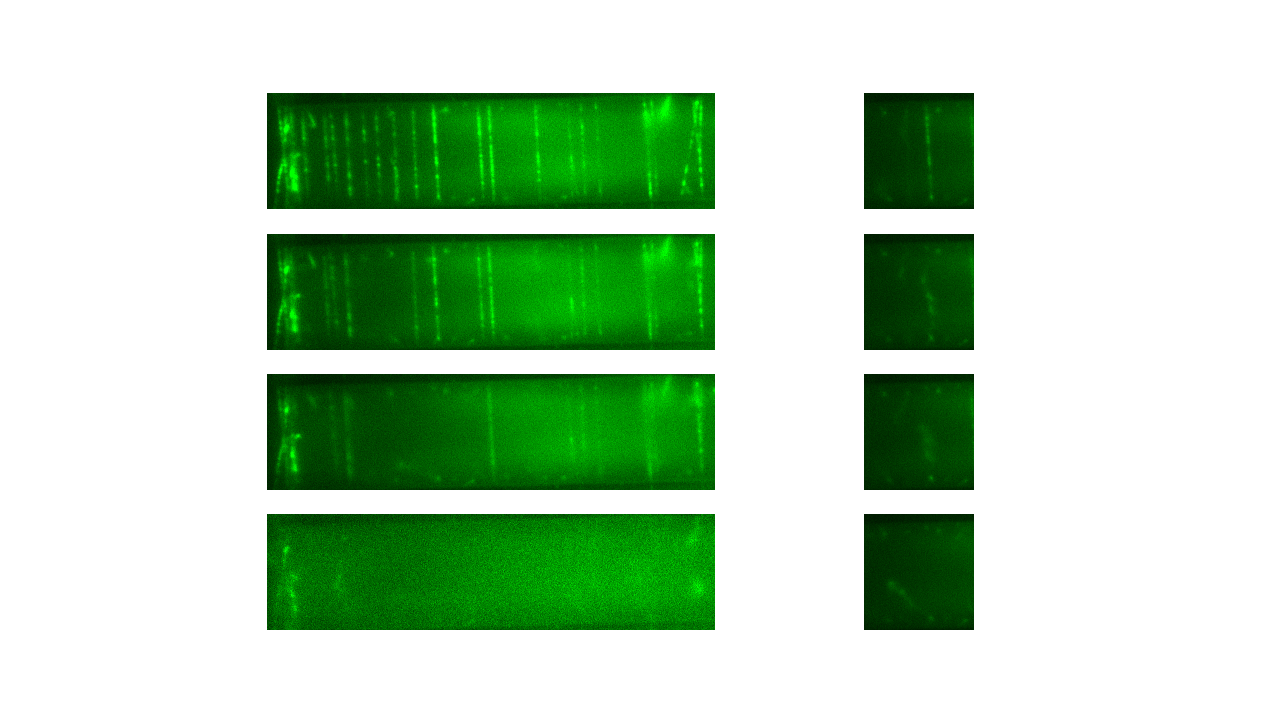

Supplement: S1 Fig — Left (from top to bottom): sequence of breakage of an ensemble of molecules. Right (from top to bottom): sequence of breakage and recoiling of a single suspended molecule. (TIF) [file pone.0260428.s001.tif]

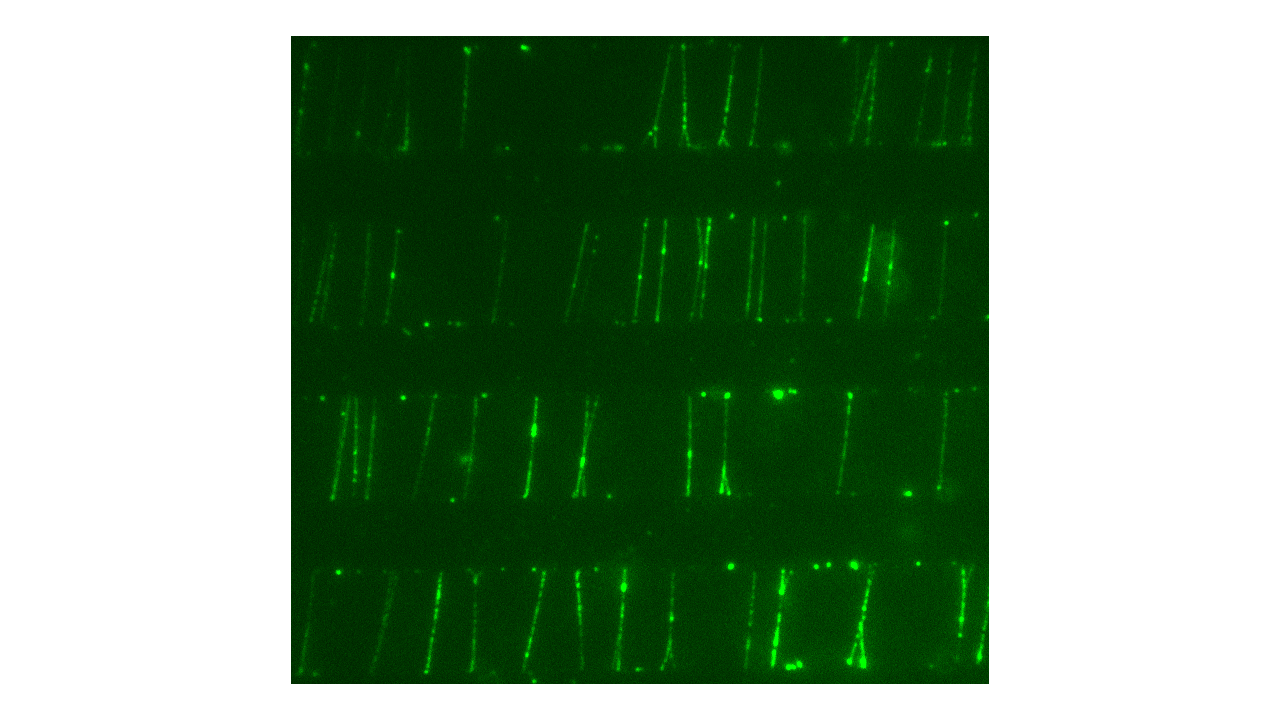

Supplement: S2 Fig — This sample was fabricated using acetone, instead of PGMEA, to dissolve the portions of SU-8 that were not crosslinked. (TIF) [file pone.0260428.s002.tif]

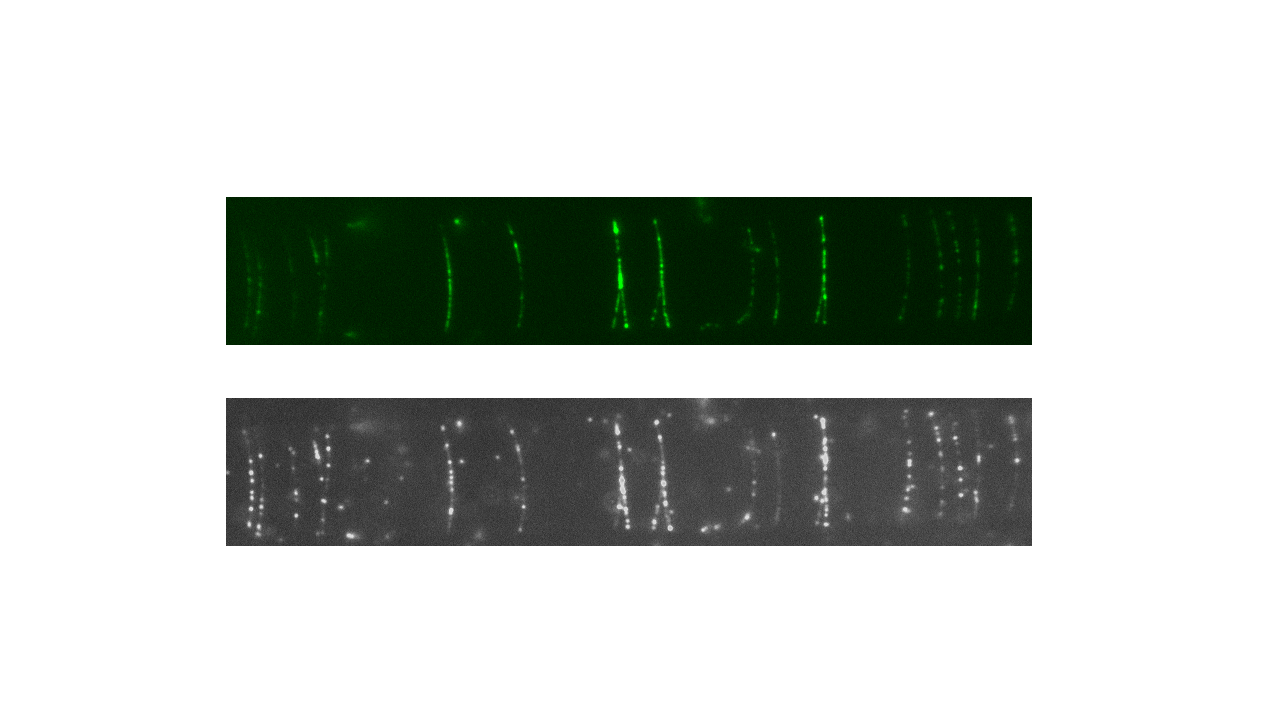

Supplement: S3 Fig — Förster resonance energy transfer (FRET) was previously used to monitor binding of a Cy5-labeled protein to nucleic acid stained with SYBR Gold [48]. We used DyLight 650 instead of Cy5, which is spectrally similar. First, we nicked the DNA bridges molecules with Nb.BtsI, then incubated the sample with biotin-11-dUTP, dATP, dCTP and polymerase. Finally, we incubated the sample with DyLight 650-labeled streptavidin. We captured an image of the sample (top) in the SYBR Gold channel. Then, we used 488 nm light to excite SYBR Gold and captured the image of the same molecules (bottom) with the emission filter (676/29) selected for DyLight 650. Note that SYBR Gold fluorescence leaks into the detection channel. (TIF) [file pone.0260428.s003.tif]
